# Numerical Simulation of the Posterior Malleolus Fracture with the Finite Element Method

**DOI:** 10.3390/jfb11010014

**Published:** 2020-03-06

**Authors:** Rafailia Ampla, Angelo V. Vasiliadis, Konstantinos Katakalos

**Affiliations:** 1 Eckersley O’ Callaghan Engineers, London WC1X 8HB, UK; rafailia@eocengineers.com; 2 PostDoc “Papageorgiou” General Hospital of Thessaloniki, 2nd Department of Orthopedic Surgery, Research associate for Laboratory for Strength of Materials and Structures, Aristotle University of Thessaloniki, 54124 Thessaloniki, Greece; vasiliadis.av@gmail.com; 3 Laboratory for Strength of Materials and Structures, Department of Civil Engineering, Aristotle University of Thessaloniki, 54124 Thessaloniki, Greece

**Keywords:** biomechanics, finite element simulation, ankle joint fractures, posterior malleolus fracture, PCL biodegradable scaffolds

## Abstract

The high demand for biodegradable implants in bone fracture fixations has dramatically increased the use of polymers for biomedical applications as well. However, the replacement of stainless steel and titanium screws by biodegradable materials represents one of the most critical aspects of biomechanics. In this study, the mechanical behavior of polycaprolactone (PCL) in tension and compression is examined. Driven by the advanced technology of computational mechanics, the fixation of the posterior malleolus fracture has been designed and analyzed. The core idea depicts the static analysis of screws made of PCL fixed in the ankle joint. The focus of the study is on this bio-absorbable, polymer-based material performance under constant compression. Parametric analysis is employed for the optimization of the PCL scaffold. Future studies will focus on the experimental verification of the numerical analysis results.

## 1. Introduction

The ankle joint is comprised of the tibia, fibula and talus. The distal fibula is called the lateral malleolus, while the distal tibia forms the plafond of the ankle joint, the medial malleolus and the posterior malleolus. Ankle fractures are among the most common type of fractures in the lower extremity. The main causes of ankle fractures are its rolling in and out, side-to-side twisting and the flexure of the joint [1,2]. Fractures of the posterior malleolus can occur in conjunction with fibular and medial malleolar fractures or in isolation [3,4]. The present research focuses on posterior malleolus fracture, which is considered to be a rare injury mechanism in comparison with lateral and medial malleolus fracture [4,5]. This fracture is located in the back of the ankle joint, while surgery for its fixation is considered necessary if the amount of joint involvement is greater than 25% and the articular step-off is greater than 2 mm [6]. The operational treatment is carried out usually with indirect reduction and stabilization with two 3.5 mm screws inserted from anterior to posterior or through a posterolateral approach using screws and/or a buttress plate [7].

To date, the most commonly used materials for this type of treatment are stainless steel and titanium, although their biocompatibility is questioned [8]. Biodegradable [9] implants made of polymers have gained ground because they are absorbed, making a second surgery for the implant’s removal unnecessary. Biodegradable scaffolds, as key artificial devices widely used in tissue engineering, aim to provide a desirable microenvironment that allows neo-tissue to be generated properly. The scaffold could provide mechanical support for the promoting neo-tissue growth and function as well as adequate porosity and permeability for nutrient delivery and metabolite removal [10]. However, as widely known among the researchers that work in the field, the main problem in scaffold design is the difference in stiffness and performance between the scaffold and the bone. Scaffolds should be porous enough to allow tissue ingrowth but not so porous that structural integrity is lost [11].

Multiobjective design optimization is essential to seek optimal scaffold architectures for promoting overall biological performance of a scaffold. Three-dimensional (3D) printing has demonstrated its great potential in producing functional scaffolds for biomedical applications. To facilitate tissue regeneration, scaffolds need to be designed to provide a suitable environment for cell growth, which generally depends on the selection of materials and geometrical features such as internal structures and pore size distribution. It is critical that the mechanical properties of the scaffold should match with those of the original tissue which is due to be repaired [8]. Nevertheless, for biodegradable scaffolds, an initial optimal design may not guarantee the ideal characteristics due to continuous material degradation and neo-tissue ingrowth [12,13].

This paper aims to explore the structural mechanism of posterior malleolus fracture and possible methods of treatment through biodegradable polycaprolactone (PCL) screws and scaffolds. Throughout this study, the finite element model which represents the fracture area is the outcome of an algorithmic process of the CT scan. As a first step, the bone is subject to static load so that the location and the value of the maximum tensile stress can be identified. A newly developed FE model, in which PCL screws are tested for the fracture fixation, is adopted here. The ability of a PCL scaffold to transfer compression loads from the broken part to the other part of the bone is further examined here. Parametric design assists the scaffold geometry identification considering the shape and volume of the fracture, as the procedure that a 3D printer would follow. A bottom line of the study is the connection type between the scaffold and the bone nodes in the finite element environment.

## 2. Methods

CT scans have become an accurate source of information for the development of finite element models, owing to the rapid development of image pre- and postprocessing., In this paper, we would like to restrain our attention on this method, and then proceed with analysis of the area where the bone fracture is and ways of fixation.

### 2.1. Three-Dimensional Finite Element Modeling from CT Data

**Figure 1:**
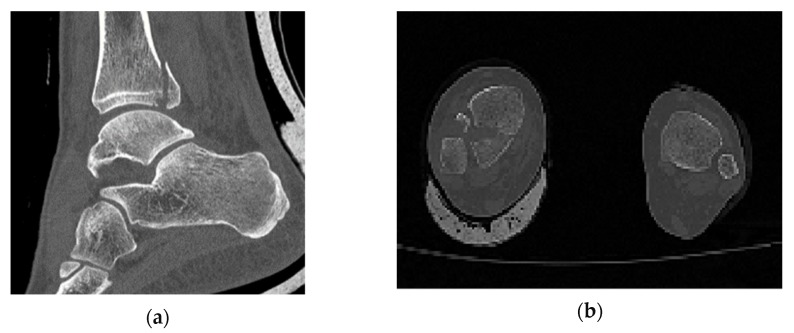
(**a**) Section cut of the posterior malleolus fracture; (**b**) plan view (fracture shown on the left).

In comparison with industrial models, which are studied through computational methods, in biomechanics the object is not designed with a CAD software and its geometry varies randomly from person to person. In order to identify that the one and only fracture is the posterior malleolus fracture, all the ankle joint parts were thoroughly observed. The software RETOMO, as a tool, gave us the opportunity to run a 3D view around the CT scan and focus further on the fracture location. In practice, the tree dimensional image is recognized through the software and converted in a surface within the boundaries that the image defines. Afterwards, the surface is meshed and replaced with face elements. The first indication that the fracture is posterior malleolus, is given as when only the tibia has a broken part and the fibula appears complete (Figure 1). A second verification is that no fracture appears on medial malleolus.

**Figure 2:**
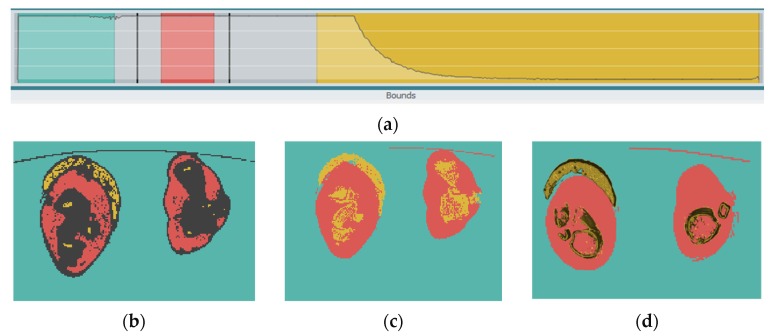
(**a**) Bounds line; (**b**) seeds algorithm output; (**c**) grow algorithm output; (**d**) Shell elements’ finite element model (FEM).

In RETOMO environment, the boundaries of different materials are not defined directly, but space is given to the user to make modifications. This is possible with the bounds line, where each material has a different color (Figure 2a). The bounds line gives the user the choice to decide where exactly the boundaries of each material are found (Figure 2b). The grey zone indicates the uncertain area (seeds algorithm). The software uses a rational path to categorize this area (grow algorithm) in different material groups. This happens by recognizing in the scan image the color and texture when the material changes. Finally, the different materials are separated, as the user has identified the limits in the bounds line (Figure 2c), which is followed by the shell elements’ finite element model (FEM) creation (Figure 2d), which is in yellow color. In pink color are the muscles and tissues highlighted as regions, as finally it is decided for them not to be modeled with finite elements.

### 2.2. Static System and Load Case

The output of RETOMO in ANSA (preprocessor) environment is composed of shell elements which form the model’s envelope. The solid mesh is achieved with tetra rapid elements—shell elements with four nodes—as a unique solution because of the bone geometry complexity. Preliminary mesh sensitivity analysis was occurred in order to define the size of elements. The bone itself could be characterized as a dynamic tissue which adapts its morphology according to the load application. In this study, the simplest static system to simulate this bone stability is adopted.

**Figure 3:**
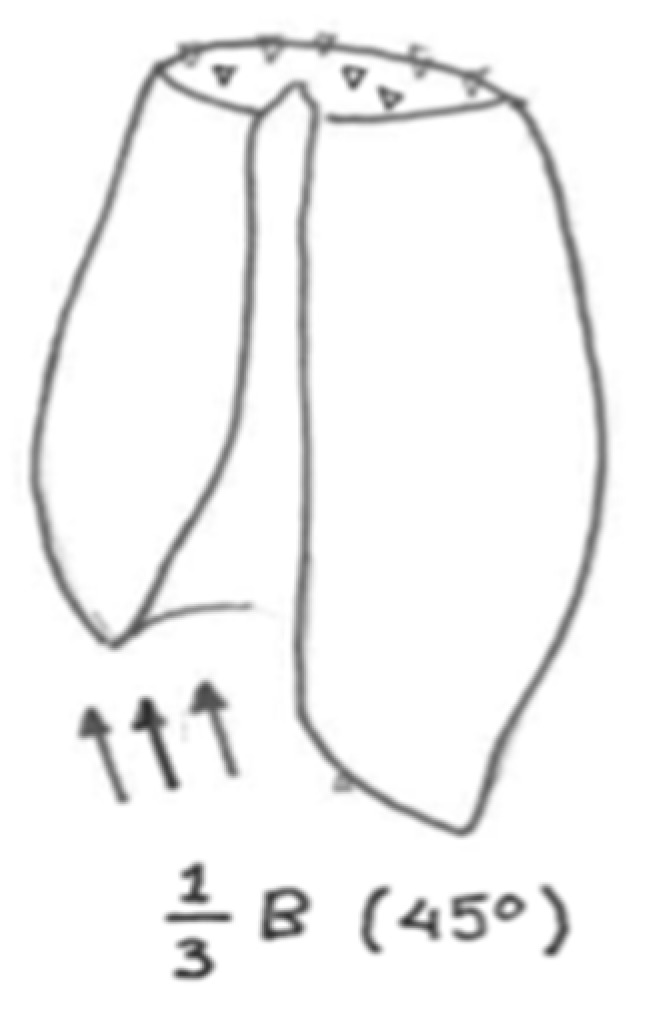
Conceptual sketch for loading and supporting the investigated fracture.

The supporting system is defined in two parts. Initially, simple supports are applied for all directions (X, Y, Z) on the top surface of the part, as depicted in Figure 3. Additionally, a simple support is applied at a random node at the bottom-right of the model for numerical stability [14]. There are three types of load applied on the broken part: weight reaction, muscles surface supporting loads and pressure due to blood flow. As the weight reaction load is the most dominant, it is chosen for this simulation. With the assumption that the average weight of a person “B” is 70 kg (700 N), each leg is allocated half of this (350 N). As for volume distribution, it is considered that one-third of it goes to the broken part, which, factored as conservative load, comes to 250 N. The load is applied as distributed in 45 degrees of the Z axis in the ZX plane. The static load case was considered appropriate to provide a sensibility check for the system’s response. To justify this engineering decision further, it could be stated that the failure under a lower static load indicates that the system would fail for sure in the combination with a higher dynamic critical load.

### 2.3. Fixation with Screws

**Figure 4:**
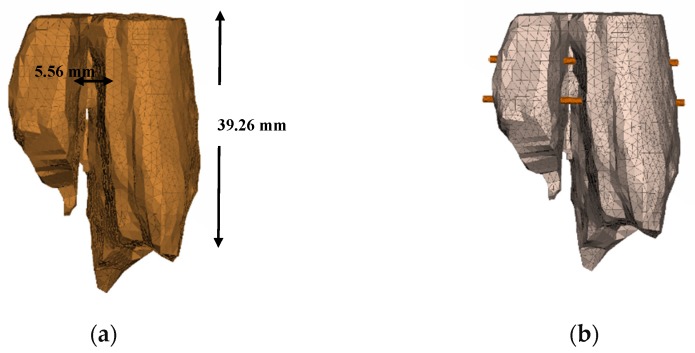
(**a**) Solid-meshed FEM; (**b**) model with screw fixation.

So that stabilization of the fracture can be achieved, the screws are fixed aiming to defend any applied action (Figure 4). Screws are often subject to torsion, shear and tension, and their assembly is in such a way to ensure a compression situation such that the fracture can be gradually closed. As a solution of this study, the fixation with two screws of 2 mm each is considered due to the small volume of the broken part, whereas for tibia fixation screws of 3–4 mm diameter are most commonly used [15].

### 2.4. Parametric Design of the Scaffold

**Figure 5:**
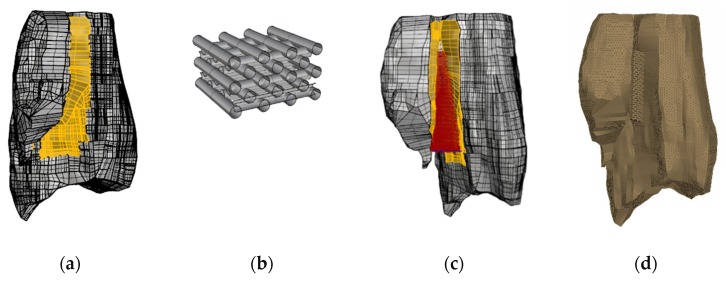
(**a**) Highlighted area of fracture in Rhino; (**b**) base geometry of scaffold; (**c**) fracture filled with scaffold material; (**d**) merged FEM in ANSA.

Parametric design can be defined as a method of problem analysis by studying more than one solution in parallel. Through this research, geometric parametric design is applied. In more detail, the shape of the bone, taken as a model, is exported from ANSA to Rhino (3D CAD software), and the area of the fracture is highlighted (Figure 5). Through the parametric design tool Grasshopper, a script is written so that this area can be filled with the scaffold material. In further detail, Grasshopper is a plug-in of Rhino. First, the user inserts the geometry of the bone in Rhino. The area where the implement is intended to be placed is highlighted. In Grasshopper environment, a script is run to recognize the boundaries. The boundaries are set, and afterwards the properties of the implant are introduced for the geometry to be developed. A tolerance of 3 mm has been intentionally left for the connection elements between the two models. The elements were rigid beams RBE2 node to node.

## 3. Results and Discussion

Bones consist of two different parts, which are the cortical (hard) bone and trabecular (spongy) bone and show different mechanical properties [16,17]. Additional bone strength is proved to be different for longitudinal and transverse loading. For the verification of the results of this study, it is supposed to be homogenized (Table 1). For the tensile and compressive strength mean values, averages of cortical longitudinal and cortical transverse have been considered.

### 3.1. Posterior Malleolus Fractures

The incidence of the posterior malleolus fractures was reported to be 7% to 44% of all ankle fractures. These types of fractures classically include the Volkmann tubercle of the posteromedial tibial plafond [18,19]. Biomechanical studies suggest that the posterior malleolus serves as an important contributor to the stability of ankle joint, while the indication of surgical treatment remains controversial [20]. The decision about surgical treatment of the posterior malleolus is traditionally based on the amount of joint involvement and articular step-off. Larger fragments involving >25% of the tibial plafond with >2 mm of articular step-off require surgical treatment in order to prevent joint instability and post-traumatic arthritis of the ankle joint [20].

### 3.2. Mechanical Behaviour of the Bone

**Figure 6:**
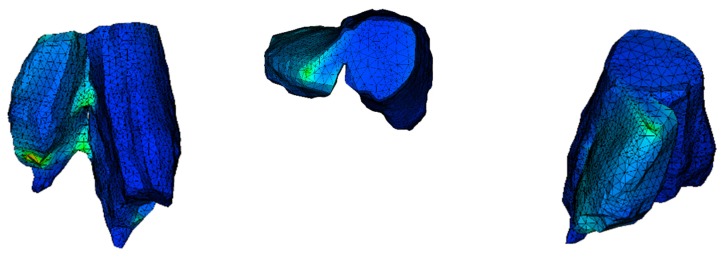
Dislocation of the bone in different perspectives (scale factor 1000).

**Figure 7:**
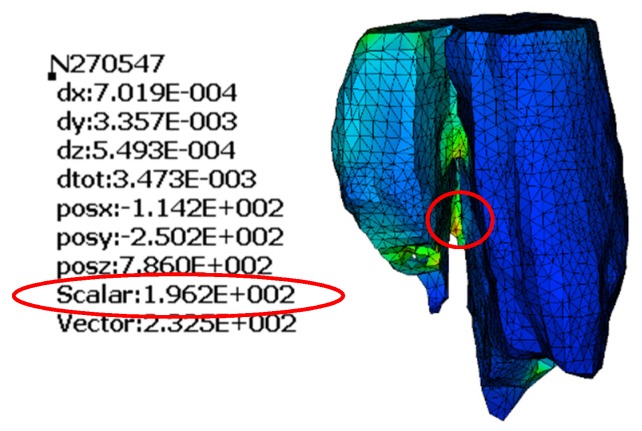
Maximum von Mises stress in MPa.

The model of the bone with no treatment fixation applied is delivered to unveil where the maximum stress is concentrated and the dislocation of it under action. It is common in finite element models for the stress to be concentrated where the load is applied. To find and point out the maximum stress, ignoring this local phenomenon, we hide those few elements. The maximum von Mises stress is 196.2 MPa and is found where the two parts of the examined bone are connected. The lack of stability in the system shows the necessity of fixation. The way that the bone tents to split (Figure 6) indicates how the treatment solution should be applied, resisting the separation of the two bone parts. The position where the higher stress is found is highlighted (Figure 7).

### 3.3. Analysis of the PCL Screws

Screws are inserted from the broken part, run through the bone and finally fixed on the other edge of the bone. The screw is not simulated in detail with the thread part. On the other hand, the shape of the screw is cylindrical, and it is connected with the bone with ‘’node to node’’ connection type. This type of connection finds each node of the solid screw cylindrical model to be in touch with a node of the solid model of the bone. The simulation is achieved by extruding a solid part of the bone depicting the volume of the screw and in fact replacing it by the screw material. The surfaces of the two members in the positions of connection are in touch. The maximum is stress is captured on one of the two screws, whereas the stress of the bone is less in comparison with the previous analysis. The maximum von Mises stress is 207 MPa, greater than the tensile strength of PCL (Table 2). This way, this type of fixation would not be considered as a possible treatment method. The stress of the other screw is 100 MPa, a result that make us think that maybe an alternative positioning of the screws combined with larger diameter could give the desirable results.

### 3.4. Analysis of the PCL Scaffold

**Figure 8:**
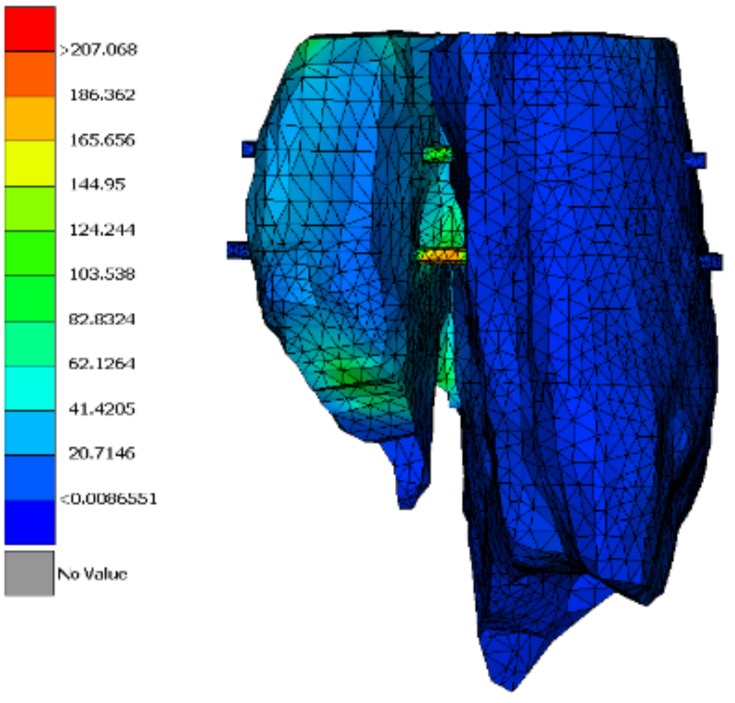
Maximum von Mises stress in MPa.

Stress distribution appears to be different from the previous two simulations. The display tool is changed to stress on node instead of stress on element. Additionally, as the mesh was quite fine in this analysis, it is not shown. The simulation of the scaffold follows the exact geometry of the implant as defined in Rhino software. The intent is to visualize the stress distribution in the scaffold model in order to improve its design. A simplified approach could be followed by applying the homogenized approach. Stress can be transferred from the broken part to the other one through the scaffold (Figure 8). The maximum stress is greater than the strength of the scaffold (Table 3). According to these analysis results, a PCL scaffold could not be a treatment solution for a fracture like this, although it could possibly have a beneficial impact if it was combined with screws. The core function of a scaffold like this is the acceleration of cell growth.

## 4. Conclusions

The numerical parametric study has shown that:

▪ In order to claim that biodegradable, polycaprolactone (PCL) materials could be used as alternative material implants, further studies should be carried out to identify their design.
▪ The use of scaffolds could be extended to a structural part in order to obtain better stress distribution on the fracture.
▪ Neither the PCL screws nor the PCL scaffold, as designed for this research, would be considered as possible fixation solutions for posterior malleolus fracture.
▪ It has to be clarified that the presented research is an initial effort and further study for geometry and location optimization is strongly encouraged, so that the optimal stress distribution could be achieved.

The next step of the research is the simulation of the dynamic–impact load that the fracture should undertake. Additionally, the research around the hybrid behavior of the PCL scaffold and titanium screws is strongly encouraged and may lead to interesting results for the fracture treatment.

## Figures and Tables

**Table 1:** Mechanical properties of bone [14].

Bone Type	E	v	Ft (MPa)	Fc (MPa)
Trabecular	350 MPa	0.25	12	30
Cortical (longitudinal)	17 GPa	0.30	109	162
Cortical (transverse)	5 GPa	0.27	50	50
Homogenized	5 GPa	0.27	80	106

**Table 2:** Mechanical properties of polycaprolactone (PCL) screws.

Member	E	v	Ft
PCL screw	0.4 GPa	0.30	80 MPa

**Table 3:** Mechanical properties of PCL scaffold [15].

Member	E (Parallel to Fibers)	E (Normal to Fibers)	v	Ft (MPa)	Fc (MPa)
PCL scaffold	138.8 MPa	37.5 MPa	0.32	5	10
